# dSTIM- and Ral/Exocyst-Mediated Synaptic Release from Pupal Dopaminergic Neurons Sustains *Drosophila* Flight

**DOI:** 10.1523/ENEURO.0455-17.2018

**Published:** 2018-06-18

**Authors:** Shlesha Richhariya, Siddharth Jayakumar, Sanjay Kumar Sukumar, Gaiti Hasan

**Affiliations:** National Centre for Biological Sciences, TIFR, Bangalore 560065, India

**Keywords:** Exo84, Neural Circuit, SOCE, Synaptic Maturation

## Abstract

Manifestation of appropriate behavior in adult animals requires developmental mechanisms that help in the formation of correctly wired neural circuits. Flight circuit development in *Drosophila* requires store-operated calcium entry (SOCE) through the STIM/Orai pathway. SOCE-associated flight deficits in adult *Drosophila* derive extensively from regulation of gene expression in pupal neurons, and one such SOCE-regulated gene encodes the small GTPase *Ral*. The cellular mechanism by which Ral helps in maturation of the flight circuit was not understood. Here, we show that knockdown of components of a Ral effector, the exocyst complex, in pupal neurons also leads to reduced flight bout durations, and this phenotype derives primarily from dopaminergic neurons. Importantly, synaptic release from pupal dopaminergic neurons is abrogated upon knockdown of dSTIM, Ral, or exocyst components. *Ral* overexpression restores the diminished synaptic release of *dStim* knockdown neurons as well as flight deficits associated with *dSTIM* knockdown in dopaminergic neurons. These results identify Ral-mediated vesicular release as an effector mechanism of neuronal SOCE in pupal dopaminergic neurons with functional consequences on flight behavior.

## Significance Statement

Appropriate wiring of neuronal circuits during development is essential for adult behavior, and cellular mechanisms such as calcium signaling orchestrate the pattern and strength of such neuronal connections to a significant extent. In *Drosophila*, flight behavior impacts multiple aspects of life. Calcium, especially through the store-operated calcium entry (SOCE) pathway, regulates gene expression during flight circuit maturation. *Ral* encodes a small GTPase and is one such SOCE-regulated gene required for *Drosophila* flight. In this paper, we show that SOCE-related loss of flight is determined to a significant extent by Ral and the exocyst complex–driven synaptic vesicle release in pupal dopaminergic neurons. Thus, SOCE-regulated release of dopamine ensures correct wiring of the flight circuit in *Drosophila*.

## Introduction

The coordinated development and maturation of neuronal circuits is essential for ensuring appropriate circuit function underlying adult behavior. Neural circuits develop and reach functional maturity through a complex process that begins with genetically encoded programs for neural cell specification and is followed by neurotransmitter fate determination, axonal growth and path finding, synapse formation, and synapse maturation (Lohmann, 2009; Blankenship and Feller, 2010). Based on the context, various calcium signaling mechanisms influence circuit development (Lohmann, 2009; Rosenberg and Spitzer, 2011), including store-operated calcium entry (SOCE) through the STIM/Orai pathway (Prakriya and Lewis, 2015). Although STIM and Orai are expressed at multiple stages (Majewski and Kuznicki, 2015), their precise role in neural development and circuit formation needs better understanding.

In the holometabolous insect *Drosophila,* the nervous system, like all other organs, undergoes metamorphosis during pupal stages to attain the adult form from the distinct larval form (Truman, 1990). Most neurogenesis is accomplished in the embryonic and larval stages followed by remodeling of existing neurons during pupal stages in tune with adult functions (Truman and Bate, 1988; Tissot and Stocker, 2000; Consoulas et al., 2002). Interestingly, attenuation of STIM/Orai-mediated SOCE in pupal neurons leads to either absent or reduced flight bout durations (Agrawal et al., 2010; Pathak et al., 2015; Richhariya et al., 2017), supporting a role for SOCE during flight circuit maturation in *Drosophila*.

In non-excitable cells, SOCE regulates cellular responses by changes in gene expression (Feske, 2007). Indeed, SOCE-regulated expression of the dopamine-synthesizing enzyme tyrosine hydroxylase has previously been demonstrated in *Drosophila* pupal neurons (Pathak et al., 2015). In mouse neural progenitor cells as well, SOCE drives gene expression (Somasundaram et al., 2014). In a screen to identify SOCE-regulated genes in *Drosophila* pupal neurons, a small GTPase, Ral, was identified as a regulator of flight (Richhariya et al., 2017).

Mammalian RalA has several roles, many of which are exocyst linked, while some are not (Gentry et al., 2014). RalA regulates the releasable pool of synaptic vesicles in mammalian neurons (Polzin et al., 2002), and both RalA and RalB mediate GTP-dependent exocytosis from neuroendocrine PC-12 cells (Wang et al., 2004; Li et al., 2007). In *Drosophila,* Ral-dependent exocyst function supports membrane addition in muscle cells (Teodoro et al., 2013), and similarly, in mouse cortical neurons, it supports neurite extension (Lalli and Hall, 2005). However, dendritic and axonal arborization patterns of central dopaminergic neurons implicated in *Drosophila* flight appear normal on attenuation of SOCE (Pathak et al., 2015).

Here we have investigated an alternate exocyst-dependent cellular mechanism by which SOCE-regulated Ral expression could help in maturation of the *Drosophila* flight circuit during pupal development. We show that release of synaptic vesicles in maturing pupal neurons requires the SOCE component *dSTIM* as well as Ral-exocyst function. We propose that dSTIM- and Ral/exocyst-dependent vesicular release is required for synaptic maturation of the flight circuit.

## Materials and Methods

### Fly rearing and stocks

*Drosophila* strains were grown on cornmeal medium supplemented with yeast. For all experiments, flies of either sex were used. For all experiments, unless stated otherwise, egg laying was performed at 25°C. Late third instar larvae were moved to 29°C to increase the expression of GAL4 and were maintained at the elevated temperature until adults eclosed, after which they were moved back to 25°C. For experiments involving GAL80^ts^, knockdown of *Exo84* or expression of *Ral^DN^* was achieved at specific developmental stages by raising the temperature to 29°C while maintaining them at 18°C for the remaining life cycle. For experiments with *dTrpA1*, flies were maintained at 22°C, and 0–2-h-old pupae were collected and transferred to 29°C for 72 h and returned to 22°C before eclosion. Fly strains used in this study are listed in Table 1.

**Table 1.** List of fly strains used

Fly line	Description	Source
elav^C155^ GAL4	Pan-neuronal driver	BDSC 458
OK371-GAL4	Glutamatergic driver	BDSC 26160
TH-GAL4	Dopaminergic driver	Friggi-Grelin et al., 2003a
TH-D1 GAL4	Dopaminergic subset neuron driver	Liu et al., 2012
TH-D’ GAL4	Dopaminergic subset neuron driver	Liu et al., 2012
TH-A GAL4	Hypoderm specific dopaminergic driver	Liu et al., 2012
TRH GAL4	Serotonergic driver	Sadaf et al., 2012
tubGAL80^ts^	GAL80^ts^ (2 copies) under tubulin promoter	McGuire et al., 2003
UAS-ANF::GFP	Rat ANF peptide tagged to GFP	BDSC 7001
UAS-spH	SynaptopHluorin	Ng et al., 2002
UAS-GCaMP6m	Calcium sensor	BDSC 42748
UAS-dTrpA1	Temperature sensitive cation channel	BDSC 26263
UAS-H_2_BmRFP	Nuclear RFP	Gift from Boris Egger Langevin et al., 2005
UAS-Shi^ts^	Temperature-sensitive Dynamin mutant	Kitamoto, 2001
UAS-Ral^DN^	Dominant negative form of Ral	BDSC 32094
UAS-Ral^IR^	RNAi against Ral	BDSC 29580
UAS-dStim^IR^	RNAi against dStim	VDRC 47073
UAS-Ral^WT^	Wild-type Ral	Richhariya et al., 2017
UAS-Exo84^IR^	RNAi against Exo84	VDRC 30111
UAS-Exo84^IR^ (line 2)	RNAi against Exo84	BDSC 28712
UAS-Sec5^IR^	RNAi against Sec5	VDRC 28874
UAS-Sec6^IR^	RNAi against Sec6	VDRC 105836
UAS-Sec6^IR^ (line 2)	RNAi against Sec6	VDRC 22079

### Single flight assay

3–5-d-old flies of either sex were anaesthetized on ice for ∼2 min and then tethered between the head and the thorax using clear nail polish on a thin metal wire. On recovery, they were given an air puff to initiate flight. The duration of flight (up to 15 min) was recorded for each fly in batches of 5–10 flies. Flight times of individual flies are represented as box plots.

### Pupal neuronal culture

24–48-h-old pupal CNSs were dissected and enzymatically digested with 50 units/ml papain (Sigma-Aldrich) activated by 1.32 mm cysteine (Sigma-Aldrich). They were then washed with DDM2 ([DMEM/F-12 with GlutaMAX (Gibco) supplemented with 100 units/ml penicillin-streptomycin (Gibco), 10 µg/ml Amphotericin B (Gibco), 20 mm HEPES (Sigma-Aldrich), 50 µg/ml insulin (Sigma-Aldrich), and 20 ng/ml progesterone (Sigma-Aldrich)], triturated with a pipette, and plated in 200 µl DDM2 for ∼4 CNS per plate coated with 0.1 mg/ml poly-d-lysine (Sigma-Aldrich). Cultures were incubated in an incubator at 25°C with 5% CO_2_ for 18–24 h before imaging.

### Live imaging for vesicular release using ANF::GFP

Pupal cultures washed and imaged in HL3 (70 mm NaCl, 5 mm KCl, 20 mm MgCl_2_, 10 mm NaHCO_3_, 5 mm trehalose, 115 mm sucrose, 5 mm HEPES, pH 7.2) with calcium (1.5 mm). Images were taken as a time series on an *XY* plane at an interval of 4 s using a 40× objective with an NA of 1.4 on an Olympus FV1000 inverted confocal microscope (Olympus Corp.). The raw images were extracted using Fiji (Schindelin et al., 2012), and regions of interest (ROIs) representing cells were selected using the Time Series Analyzer plugin. Percentage Δ*F*/*F* release was calculated as (*F*_0_ – *F_t_*)/*F*_0_ × 100, where *F_t_* is the fluorescence at time *t* and *F*_0_ is baseline fluorescence corresponding to the average fluorescence over the first 10 frames. The area under the curve from these response curves was calculated from 300 to 900 s using Microsoft Excel (Microsoft) and implementing the trapezoidal rule (Nedelman and Gibiansky, 1996; Qin et al., 2009) where

$$AUC=\int_{t1}^{t2}\;\frac{\Delta F}{F}\;(t)\partial t$$

Here, t_1_ = 300 s and t_2_ = 900 s, and this accounts for both positive and negative AUC measurements. For experiments involving *UAS-Shi^t^* ^s^, a heated microscope stage was used.

### Ex vivo synaptopHluorin imaging from pupal brains

Brains were dissected in larval hemolymph-like saline (HL3) consisting of 108 mm NaCl, 5 mm KCl, 2 mm CaCl_2_, 8.2 mm MgCl_2_, 4 mm NaHCO_3_, 1 mm NaH_2_PO_4_, 5 mm trehalose, 10 mm sucrose, 5 mm Tris, pH 7.5, and filtered through a 0.2-μm filter (Makos et al., 2010). Dissected brains expressing synaptopHluorin (spH) were embedded in 0.2% low-melt agarose and bathed in HL3. Images were taken as a time series across depth at an interval of 4 s using a 10× objective with an NA of 0.4 on a Leica SP5 confocal microscope using a resonant scanner allowing for fast scan speeds. Images were analyzed using Fiji. The *XYZ* time series was projected along *Z* using the maximum-intensity function on Fiji. ROIs were selected using the Time Series Analyser plugin. Δ*F*/*F* was calculated as (*F_t_* – *F*_0_)/*F*_0_, where *F*_0_ is baseline fluorescence corresponding to the average fluorescence over the first 10 frames and *F_t_* is the fluorescence at time *t*. Area under the curve was calculated from 60 to 300 s for saline-induced release and from 300 to 600 s for evoked release by standard methods invoking the trapezoid rule as mentioned above using Microsoft Excel.

### Data representation and statistics

All flight data and quantifications of vesicular release are represented as box plots using BoxplotR (Spitzer et al., 2014), where horizontal lines represent medians, crosses indicate means, box limits indicate 25th and 75th percentiles, whiskers extend 1.5 times the interquartile range from the 25th and 75th percentiles, individual data points are represented as open circles, and the numbers below represent the *n* number for each box. Traces from live-imaging experiments represent means (±standard error of means) from all cells or ROIs.

Comparisons between two samples were performed using the two-tailed Student’s *t* test. If more than two conditions were involved, one-way ANOVA followed by Tukey’s test was used. All statistical tests were performed using Origin 8.0 software (Micro Cal). Statistical tests and exact *p*-values used in each figure are listed in Table 1-1.

10.1523/ENEURO.0455-17.2018.t1-1 Table 1-1 Statistical tests and *p*-values for all figures. Download Table 1-1, XLSX file.

## Results

### Exocyst function during pupal development is required for adult flight maintenance

**Figure 1.**
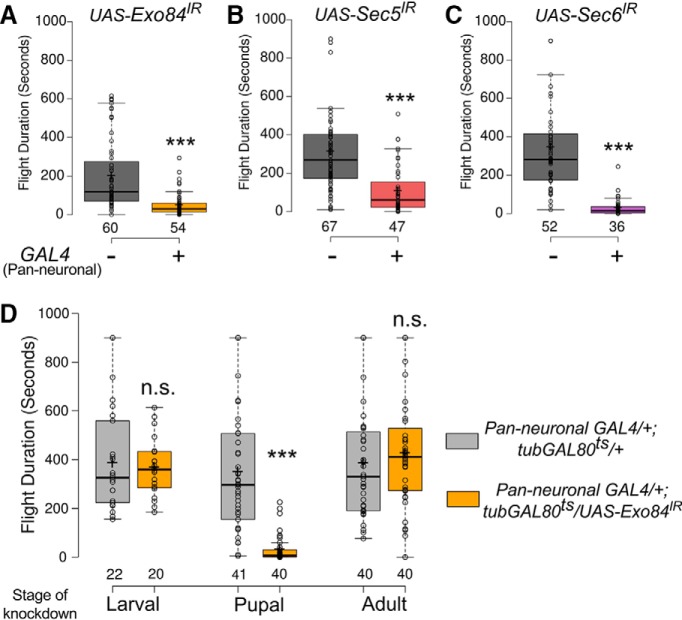
Exocyst components are required in pupal neurons for maintaining the duration of adult flight bouts (refer to Figs. 1-1 and 1-2 and Table 1-1). ***A–C***, Box plots represent durations of flight bouts in flies from the indicated genotypes measured by the single flight assay. ***D***, Box plots represent durations of flight bouts in flies with knockdown of Exo84 in neurons during the indicated stages of development. In the box plots, horizontal lines represent medians, crosses indicate means, box limits indicate 25th and 75th percentiles, whiskers extend 1.5 times the interquartile range from the 25th and 75th percentiles, individual data points are represented as open circles, and the numbers below represent the n number for each box. ***, p < 0.001, n.s., not significant at p < 0.05 by two-tailed Student’s t test. For exact p-values, refer to Table 1-1.

Inhibition or knockdown of the small GTPase Ral in pupal neurons leads to significant flight deficits in adult flies (Richhariya et al., 2017). To test if Ral function in pupal neurons is exocyst dependent, three components of the exocyst complex, Sec5, Sec6, and Exo84 (Finger and Novick, 1998; Guo et al., 1999) were independently knocked down by pan-neuronal expression of RNA interference constructs with the GAL4 driver *Elav^C155^* (Lin and Goodman, 1994). Knockdown of each of the three exocyst components resulted in significantly shortened flight durations compared to the corresponding RNAi controls (Fig. 1*A–C*). This indicates a role for the exocyst complex in flight, as none of the RNAis used have any predicted off-targets (Dietzl et al., 2007). Furthermore, significant flight defects were also obtained with independent RNAi lines for *Exo84* and *Sec6* (Fig 1-1*A*,*B*).

10.1523/ENEURO.0455-17.2018.f1-1 Figure 1-1 The role of exocyst components in flight. ***A***, ***B***, Box plots represent flight durations of flies of the indicated genotypes. Knockdown of *Exo84* and *Sec6* with alternate RNAi lines resulted in similar phenotypes as shown in Fig. 1. These flies were grown at 25°C throughout. ***C***, ***D***, Box plots represent flight durations of control flies with RNAi-mediated knockdown of *Exo84* (*Exo84^*IR*^*) in neurons raised at the indicated temperatures throughout development. At the permissive temperature of 18°C, there is no knockdown. *GAL4*-mediated knockdown of *Exo84* occurs at the restrictive temperature of 29°C. Box plot symbols are as described in Fig. 1. *, *p* < 0.05, **, *p* < 0.01, ***, *p* < 0.001, n.s., not significant at *p* < 0.05 by two-tailed Student’s *t* test (for ***A*** and ***B***) or one-way ANOVA followed by *post hoc* Tukey’s test (for ***C*** and ***D***). All comparisons for significance were with the control values except where marked by a horizontal line. For exact *p*-values, refer to Table 1-1. Download Figure 1-1, TIF file.

**Figure 2.**
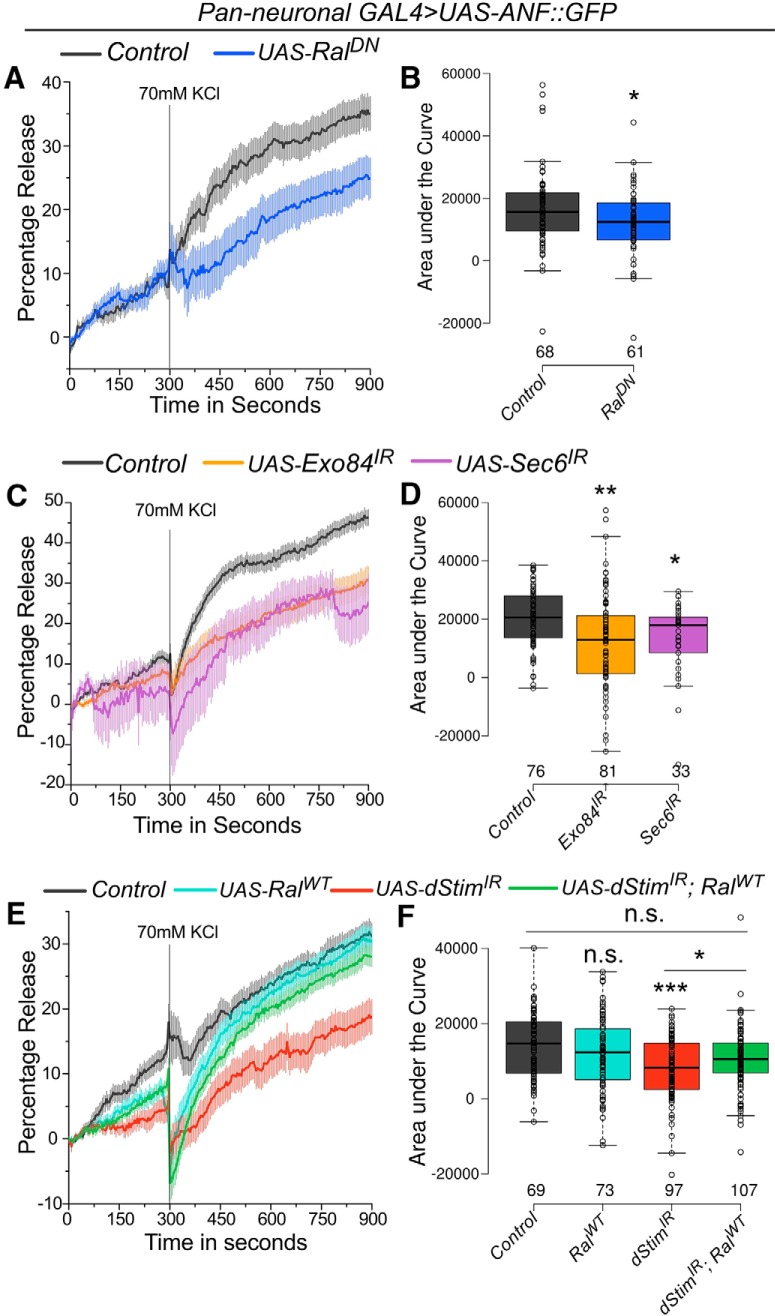
*dStim* regulates vesicular release in pupal neurons through Ral (refer to Fig. 2-1 and Table 1-1). ***A***, ***C***, ***E***, Traces represent release of ANF::GFP, estimated by reduction in GFP fluorescence, on depolarization with KCl. Lines represent means and the error bars, standard error of means, from cells as mentioned in the box plots on the right. Point of KCl addition is indicated with a line. ***B***, ***D***, ***F***, Amount of release quantified as area under the curve from 300 to 900 s is represented as box plots for the indicated genotypes. Box plots symbols are as described in Fig. 1. Numbers below each box represent number of cells analyzed from at least three different culture dishes. *, *p* < 0.05, **, *p* < 0.01, ***, *p* < 0.001, n.s., not significant at *p* < 0.05 by two-tailed Student’s *t* test (for ***A***) or one-way ANOVA followed by *post hoc* Tukey’s test (for ***D*** and ***F***). All comparisons for significance were with the control values except where marked by a horizontal line. For exact *p*-values, refer to Table 1-1.

10.1523/ENEURO.0455-17.2018.f1-2 Figure 1-2 Expression of exocyst components is not regulated by SOCE. ***A–D***, Bar graphs represent the level of expression of the indicated genes using values for fragments per kilobase gene per million reads (FPKM) in control and *dStim* knockdown pupal brains. These data were obtained from the transcriptomic screen performed in Richhariya et al. (2017). *q*-values are as obtained by CuffDiff. Download Figure 1-2, TIF file.

The temporal requirement of the exocyst complex for flight was investigated by knockdown of *Exo84*, as a proxy for the complex, at different developmental stages using the TARGET system (McGuire et al., 2004). Knockdown in the pupal stage alone resulted in flight deficits equivalent to those observed by knockdown throughout development, suggesting that *Exo84* expression is exclusively required during pupal development for maturation of the flight circuit. Indeed, *Exo84* knockdown exclusively in either the larval or adult stage did not alter flight durations significantly from the respective controls (Figs. 1*D* and 1-1*C*,*D*). This temporal requirement of *Exo84* in the context of flight coincides with that of Ral and SOCE observed earlier (Richhariya et al., 2017). Therefore, regulation of exocyst components by SOCE was tested next. There was no change in mRNA levels of the three exocyst components, *Exo84*, *Sec5*, and *Sec6*, in pupal neurons with knockdown of a core SOCE component, the ER-Ca^2+^ sensor, *dStim*, when Ral levels were found to be reduced (Richhariya et al., 2017; Fig. 1-2*A–D*). These data demonstrate a requirement for SOCE, Ral, and *Exo84* in pupal neurons for adult flight maintenance and suggest that SOCE modulates exocyst-dependent vesicular release in maturing pupal neurons, most likely by regulating Ral expression as identified earlier (Richhariya et al., 2017).

### Ral functions downstream of *dStim* to mediate vesicular release in pupal neurons

To measure vesicular release in pupal neurons, we used the rat atrial natriuretic peptide tagged with emerald GFP (ANF::GFP). ANF::GFP is recognized by the *Drosophila* protein-sorting machinery, and its expression is detected in all neurons after embryonic stage 17 when driven by a pan-neuronal GAL4 (Rao et al., 2001). Vesicular release by ANF::GFP expression has been measured from peptidergic (Kula et al., 2006) and nonpeptidergic (Levitan et al., 2007; Liu et al., 2012) neurons *in vivo*. The vesicular release assay in pupal neurons was standardized by expression of a temperature-sensitive dynamin mutant, *Shibire^ts^* (*Shi^ts1^*), that reversibly blocks vesicular release in *Drosophila* neurons at 29°C but not at 22°C (Kitamoto, 2001) and can also block peptide release at the restrictive temperature (Wong et al., 2015). In neurons expressing both ANF::GFP and *Shi^ts1^*, depolarization by KCl at 22°C resulted in a decrease in ANF::GFP intensity at the soma of cultured neurons over time (Fig. 2-1*A–C*). To rule out effects of elevated temperature on neuronal activity–mediated release (Tomchik, 2013), or on GFP fluorescence, we performed the same experiment without *Shi^ts1^* at 29°C and observed release comparable to that at the permissive temperature of 22°C with *Shi^ts1^* (Fig. 2-1*B*,*C*). The small rise observed just before stimulation could be either basal release or possibly an artifact of photobleaching, as it was similar under all conditions (Fig. 2-1*B*,*D*). Furthermore, the drop in intensity of ANF::GFP was specific to a depolarization stimulus and not due to osmotic shock, because replacement of KCl with NaCl (at the same concentration as KCl) did not result in reduction of ANF::GFP intensity above baseline levels (Fig. 2-1*C*,*D*). Thus, we interpret reduction in GFP intensity after KCl addition as vesicular release from the soma due to depolarization. Blocking vesicular recycling by subjecting neurons expressing *Shi^ts1^* to the restrictive temperature (29°C) while imaging led to a significant reduction in release of ANF::GFP compared to the permissive temperature (Fig. 2-1*A–C*), validating this assay for assessing vesicular release in primary cultures of pupal neurons.

Inhibiting Ral function in cultured pupal neurons by expression of a dominant-negative form of Ral, *Ral^DN^,* led to a significant reduction in release of ANF::GFP (Fig. 2*A*,*B*). A similar reduction in release was observed on knockdown of either *Exo84* or *Sec6* (Fig. 2*C*,*D*), which correlates with the strong flight defects observed on their knockdown (Fig. 1*A*,*C*). Knockdown of an essential neuronal SOCE component, *dStim*, also led to reduction in ANF::GFP release, as predicted from previous data demonstrating that Ral expression in pupal neurons requires SOCE (Richhariya et al., 2017, Fig. 2*E*,*F*). Furthermore, reduced release of ANF::GFP by *dStim* knockdown could be rescued by overexpressing *Ral^WT^* (Fig. 2*E*,*F*), indicating that Ral function is downstream of SOCE in mediating vesicular release in pupal neurons.

10.1523/ENEURO.0455-17.2018.f2-1 Figure 2-1 Assay to measure vesicular release from cultured pupal neurons. ***A***, Representative images of single somata of cultured pupal neurons expressing ANF::GFP and *Shi^*ts*^*. The images were obtained over time upon depolarization with KCl at permissive (22°C) and restrictive (29°C) temperatures. Scale bar represents 10 µm. ***B***, ***D***, Traces represent release of ANF::GFP, estimated by the reduction in GFP fluorescence, upon depolarization with KCl, or addition of NaCl over time. The control trace in ***D*** is the same as that in Fig. 2*A*. ***C***, ***E***, Amount of release quantified as area under the curve from 300 to 900 s is represented as box plots for the indicated genotypes. Box plot symbols are as described in Fig. 1. ***, *p* < 0.001, n.s., not significant at *p* < 0.05 by one-way ANOVA followed by *post hoc* Tukey’s test (for ***C***) or two-tailed Student’s *t* test (for ***E***). All comparisons for significance were with the control values except where marked by a horizontal line. For exact *p*-values, refer to Table 1-1. Download Figure 2-1, TIF file.

### Ral function in pupal dopaminergic neurons is required for adult flight maintenance

**Figure 3.**
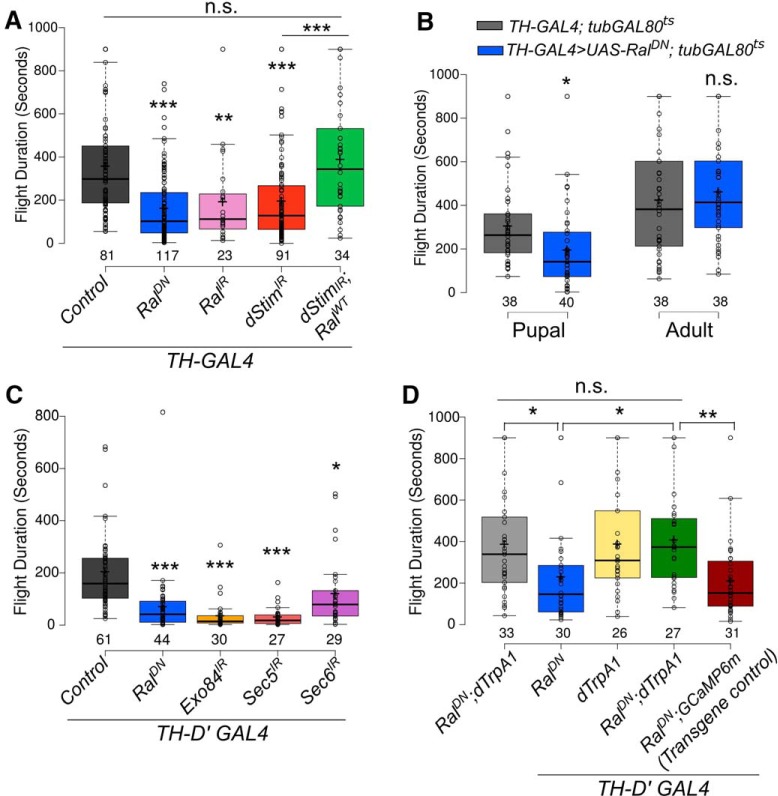
Extended flight bout durations in adults requires Ral and exocyst function in pupal dopaminergic neurons (refer to Fig. 3-1 and Table 1-1). ***A***, ***C***, Box plots represent flight bout durations of flies from the indicated genotypes measured by the single flight assay. ***B***, Box plots represent flight bout durations of flies with expression of a dominant negative mutant of Ral (*Ral^DN^*) in dopaminergic neurons during the indicated stages of development. ***D***, Box plots represent flight bout durations of flies from the indicated genotypes that were grown at 22°C and transferred to 29°C from 0 to 72 h after Puparium formation for activation of *dTrpA1*. Box plot symbols are as described in Fig. 1. *, *p* < 0.05, **, *p* < 0.01, ***, *p* < 0.001, n.s., not significant at *p* < 0.05 by two-tailed Student’s *t* test (for ***B***) or one-way ANOVA followed by *post hoc* Tukey’s test (for ***A***, ***C***, and ***D***). All comparisons for significance were with the control values except where marked by a horizontal line. For exact *p*-values, refer to Table 1-1.

Next, we tested the spatio-temporal requirement for Ral in previously identified neuronal subsets where loss of SOCE is known to affect the maintenance of flight bouts. These include dopaminergic (Pathak et al., 2015) and glutamatergic (Venkiteswaran and Hasan, 2009) neurons. Blocking Ral function either in all glutamatergic neurons by the *OK371-GAL4* (Mahr and Aberle, 2006) or in the motor neurons using the *OK6-GAL4* (Sanyal, 2009) resulted in significantly reduced flight durations (Fig. 3-1*A*,*B*). Similarly, reducing either Ral levels or function in dopaminergic cells, with *TH-GAL4* (Friggi-Grelin et al., 2003a), resulted in significantly reduced flight durations that were comparable to flight deficits observed by knockdown of *dStim* (Fig. 3*A*). Importantly, the flight deficits observed on *dStim* knockdown were rescued to control levels by restoring Ral function, when *Ral^WT^* was overexpressed in this background (Fig. 3*A*). *Ral* knockdown exclusively during the pupal stage, but not in the adult stage, resulted in flight durations significantly lower than those of control flies (Figs. 3*B* and 3-1*C*,*D*).

10.1523/ENEURO.0455-17.2018.f3-1 Figure 3-1 Neuronal subsets where Ral function is required for maintenance of flight bouts. ***A–G***, Box plots represent flight durations of flies from the indicated genotypes measured by the single flight assay. Box plot symbols are as described in Fig. 1. Statistically distinguishable groups are indicated by different alphabets over the box plots (two-tailed Student’s *t* test, *p* < 0.05). *, *p* < 0.05, **, *p* < 0.01, ***, *p* < 0.001, n.s., not significant by two-tailed Student’s *t* test. For exact *p*-values, refer to Table 1-1. Download Figure 3-1, TIF file.

Because *TH-GAL4* expresses in both neuronal and non-neuronal cells (Friggi-Grelin et al., 2003b), we further refined the spatial requirement of Ral for flight using GAL4 drivers that mark dopaminergic cell subsets. Significantly shortened flight durations were observed on reducing Ral function with the neuronal-specific *TH-D′* (Fig. 3*C*) and *TH-D1* (Fig. 3-1*F*) *GAL4s* (Liu et al., 2012; Pathak et al., 2015). However, attenuating Ral function in hypodermal cells with *TH-A GAL4* (Liu et al., 2012) did not significantly alter the duration of flight (Fig 3-1*G*). Blocking Ral function in serotonergic neurons with the *TRH-GAL4* also did not affect flight duration (Fig. 3-1*E*). Thus, *Ral* is required in pupal dopaminergic neurons for a functional adult flight circuit. These data are in agreement with previous findings demonstrating that loss of SOCE by inhibition of dOrai in similar subsets of pupal dopaminergic neurons impairs flight in adult *Drosophila* (Pathak et al., 2015).

Knockdown of the exocyst components, *Exo84, Sec5*, and *Sec6*, in *TH-D′GAL4*-marked dopaminergic neurons also resulted in flight deficits (Fig. 3*C*). Importantly, the flight deficits observed on reducing Ral function in dopaminergic neurons could be rescued by expression of the heat-activated cation channel *dTrpA1* in *TH-D′* marked neurons in pupae (Fig. 3*D*). Expression of a neutral transgene, GCaMP6m, had no effect on the fight deficit shown by animals expressing *Ral^DN^* in *TH-D′*-marked neurons (Fig 3*D*). dTrpA1 enhances neuronal activity (Hamada et al., 2008) and thus, possibly, the extent of vesicular release in pupal dopaminergic neurons. This was tested next.

### *dStim* regulates synaptic vesicle release from pupal dopaminergic neurons through *Ral*

Vesicular release from neurons can occur either through small synaptic vesicles (SSVs) or large dense core vesicles (LDCVs). SSVs are small, clear vesicles that primarily carry neurotransmitters whereas LDCVs are large, granular vesicles that transport neuropeptides, hormones and aminergic neurotransmitters (Park and Kim, 2009). Mammalian Ral colocalizes with both SSVs (Volknandt et al., 1993) and LDCVs (De Leeuw et al., 1999) and hence, regulation by Ral of both classes of vesicles is possible in *Drosophila*. The assay with ANF::GFP in cultured neurons (Figs. 2 and 2-1) most likely assayed for release from LDCVs at the soma, and although such vesicles can corelease a monoamine neurotransmitter like dopamine (Nusbaum et al., 2017), it would not test the ability of Ral to affect neurotransmitter release from SSVs. To measure release from SSVs at synaptic release sites in the maturing pupal brain, we used spH, a pH-sensitive GFP, that localizes to synaptic vesicles and whose fluorescence is quenched in acidic conditions but fluoresces on fusion of the vesicle with the pre-synaptic membrane (Miesenböck et al., 1998). SSV release was important to assess, because SOCE-regulated expression of TH, a dopamine-synthesizing enzyme, in pupal stages is required for adult flight (Pathak et al., 2015), suggesting that formation of appropriate functional connectivity between central dopaminergic neurons and their postsynaptic targets requires dopamine release. We hypothesized that SOCE and Ral-dependent exocyst function could be a mechanism underlying such dopamine release in developing synapses of the pupal brain.

**Figure 4.**
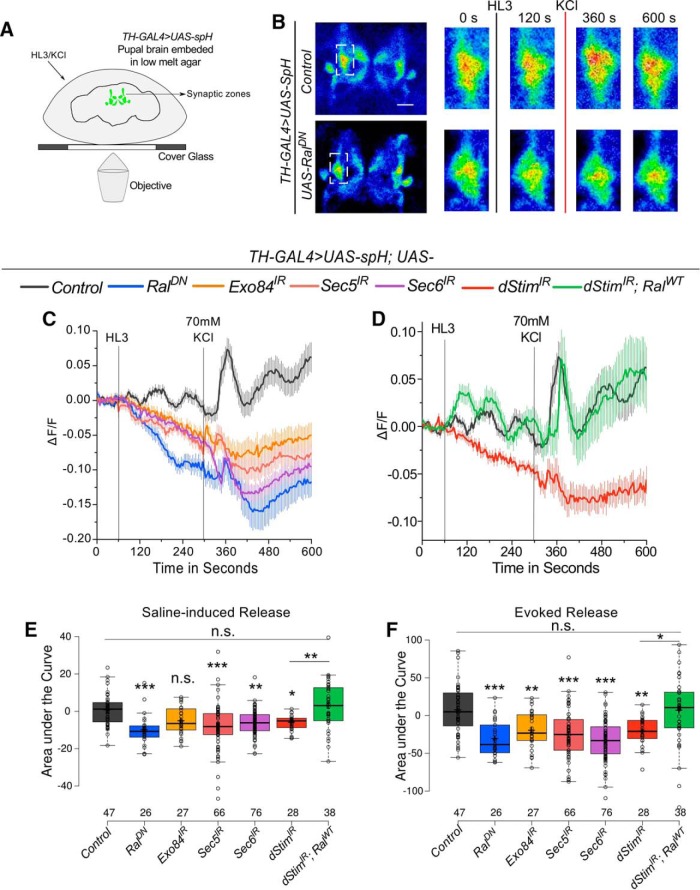
*dStim* regulates evoked synaptic release from pupal dopaminergic neurons through *Ral* (Refer to Fig. 4-1 and Table 1-1). ***A***, Schematic representation of the experimental setup used to image vesicular release from pupal brains. ***B***, Representative images of central regions of pupal brains expressing spH in dopaminergic neurons. Boxed images on the right were obtained by zooming into the inset (white box in the images on the left) at the indicated time points of the time series. ***C***, ***D***, Traces represent average (±SEM) change in fluorescence of spH over time from brains of the indicated genotypes. Points of addition of HL3 and KCl are denoted by vertical lines. ***E***, ***F***, Amount of release quantified as area under the curve from 60 to 300 s (E-Saline–induced) and 300 to 600 s (F-KCl–evoked) from traces in ***C*** and ***D*** is represented as box plots for the indicated genotypes. Box plot symbols are as described in Fig. 1. Numbers below the boxes represent number of ROIs in which fluorescence changes were measured. They were obtained from a minimum of five brains per genotype. *, *p* < 0.05, **, *p* < 0.01, ***, *p* < 0.001, n.s., not significant at *p* < 0.05 by one-way ANOVA followed by *post hoc* Tukey’s test. All comparisons for significance were with the control values except where marked by a horizontal line. For exact *p*-values, refer to Table 1-1.

Synaptic release from dopaminergic neurons in pupae was visualized in *ex vivo* preparations of 48–72-h old pupal brains expressing *UAS-spH* (Ng et al., 2002) using *TH-GAL4* (Fig. 4*A*). The *TH-GAL4* marks synaptic zones in parts of the mushroom body (MB) viz. the α and α′ vertical lobes, upper stalk, lower stalk and junction, and the distal heel and peduncle deriving from the PPL1 neurons among others (Mao and Davis, 2009). Thus, synaptic release was measured at maturing synaptic zones of the MB (Fig. 4*A*). Depolarization by KCl evoked vesicle release, as evidenced by a visible and quantifiable change in spH fluorescence (Video 1 and Fig. 4*B–D*,*F*). The amplitude and kinetics of the change in fluorescence observed were similar to what have been observed earlier using spH in mammalian (Burrone et al., 2007) and *Drosophila* neurons (Slawson et al., 2014). Interestingly, addition of hemolymph-like saline (HL3) caused a measurable level of synaptic release at the MB synaptic zones even without KCl-induced depolarization (Video 1 and Fig. 4*C*,*E*). This saline-induced release could result from basal activity of the endogenous circuit activity, because the recordings were performed from an intact live brain without any inhibitors. Both saline and depolarization evoked release from pupal dopaminergic neurons were severely affected on either, reducing Ral function or knockdown of exocyst components as well as knockdown of *dStim* (Figs. 4*B–F* and 4-1*D–F*). Importantly, overexpression of wild-type *Ral* in the background of *dStim* knockdown rescued both saline and KCl evoked release (Fig. 4*D–F*). Expression of a neutral transgene, *UAS-H_2_BmRFP* in *dStim* knockdown background, did not rescue either kind of release (Fig. 4-1*A–C*), demonstrating that rescue was indeed because of restoring *Ral* levels and not insufficient knockdown of *dStim* caused by expression of multiple transgenes.

10.1523/ENEURO.0455-17.2018.f4-1 Figure 4-1 *dStim* and *Ral* regulate synaptic release from pupal dopaminergic neurons. ***A***, ***D***, Traces represent change in fluorescence of spH over time from brains of the indicated genotypes. Lines represent means and the error bars, standard error of means. Points of addition of HL3 and KCl are denoted by vertical lines. ***B***, ***E***, Saline-induced release as quantified by the area under the curve from 60 to 300 s of the respective traces. ***C***, ***F***, Evoked release as quantified by the area under the curve from 300 to 600 s of the respective traces. Box plot symbols are as described in Fig. 1. Numbers below the boxes represent the number of ROIs measured for change in fluorescence from a minimum of five brains per genotype. *, *p* < 0.05, **, *p* < 0.01, ***, *p* < 0.001, n.s., not significant at *p* < 0.05 by two-tailed Student’s *t* test (for ***B*** and ***C***) or one-way ANOVA followed by *post hoc* Tukey’s test (for ***E*** and ***F***). For exact *p*-values, refer to Table 1-1. Download Figure 4-1, TIF file.

Video 1. Visualizing synaptic release from dopaminergic neurons. Changes in fluorescence of spH driven by the *TH-GAL4* indicative of synaptic release from dopaminergic neurons in the mushroom body. Gray flash indicates addition of HL3 (mock); red flash indicates addition of 70 mm KCl. 10.1523/ENEURO.0455-17.2018.video.1

## Discussion

**Figure 5.**
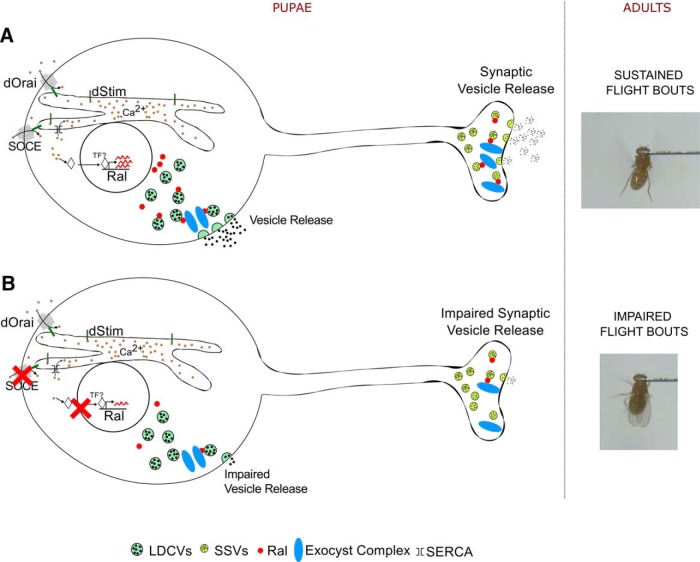
Vesicle release regulated by SOCE, Ral, and the exocyst in pupal neurons regulates flight in adults. ***A***, Cartoon representation of a pupal neuron where SOCE regulates Ral expression. Ral is required for tagging secretory vesicles toward the exocyst complex, thus marking them for both somatic and synaptic release. Such regulated vesicular release of dopamine from pupal neurons appears essential for maturation of a neural circuit required in adults for maintaining the duration of flight bouts. ***B***, In conditions of dStim knockdown, when SOCE is attenuated, Ral levels are reduced, and this in turn has a negative impact on vesicular release in dopaminergic neurons. Consequently, flight bout durations in adult flies are also reduced.

Neuronal Ral was recently identified as a regulator of *Drosophila* flight (Richhariya et al., 2017). In this study, we have identified dopaminergic neurons as a cellular focus for Ral function during flight circuit maturation in pupae. In dopaminergic neurons Ral functions through Exo84, and presumably the exocyst, to regulate synaptic release during pupal development. Moreover, we identify a novel role for the SOCE gene *dStim* in regulating synaptic vesicular release through positive control of Ral gene expression, identified previously in a screen for SOCE-regulated genes (Richhariya et al., 2017). Importantly, both flight deficits and reduced synaptic vesicular release observed as a consequence of dSTIM knockdown were rescued by Ral over-expression in dopaminergic neurons. These findings support an essential requirement for dopamine release during synapse formation and maturation of the *Drosophila* flight circuit (Pathak et al., 2015; Fig. 5).

We observed a loss of somatic and synaptic vesicular release on knockdown of *dStim*, *Ral*, and the exocyst components tested (Figs. 2 and 4). Because Ral and the exocyst have established roles in regulating pre-synaptic vesicle dynamics and exocytosis (Moskalenko et al., 2002; Wang et al., 2004) and *Ral* expression is regulated by *dStim* (Richhariya et al., 2017), we propose that *dStim* regulates vesicular release via *Ral*. However, the reduction in vesicular release on *dStim* knockdown might also be mediated by other factors such as expression levels and densities of ion channels (Voglis and Tavernarakis, 2006) and altered signaling in response to neurotransmitters (Huang and Thathiah, 2015), which have not been tested here. Because *Ral* almost completely rescues the release defects of *dStim* knockdown (Figs. 2*E*,*F* and 4*E*,*F*), we believe that a significant component of *dStim* mediated vesicular release in pupal dopaminergic neurons is by regulating expression of *Ral*.

Activity-dependent calcium entry is essential for synaptic release (Südhof, 2012). However, this is the first report where calcium entry through SOCE regulates synaptic release. Our work demonstrates an important distinction between how the two modes of calcium entry regulate synaptic vesicle release, viz., that SOCE-regulated synaptic release is indirect by regulation of Ral expression. Thus, neuromodulators that stimulate SOCE very likely change synaptic function over time scales that are much greater than activity-based synaptic release. We favor the dSTIM/dOrai mode of SOCE in this context primarily because very similar flight deficits are observed by knockdown of *dOrai* and these can also be rescued by overexpression of Ral (Richhariya et al., 2017).

Data in this paper suggest that *dStim,* by regulation of *Ral* expression (Richhariya et al., 2017), impacts both spontaneous and evoked synaptic vesicle release in pupal dopaminergic neurons. Neurotransmitter release from SSVs and neuropeptide as well as neurotransmitter release from LDCVs, regulates multiple steps during the development of neural circuits, including synapse formation and maturation (Lochner et al., 2008; Park and Kim, 2009; Tau and Peterson, 2010). Neurotransmitter release during circuit development can affect both synapse formation and strength (Bleckert and Wong, 2011). Moreover, spontaneous release of neurotransmitters, similar to an earlier observation in larval neurons (Choi et al., 2014), could be important for synaptic maturation. The absence of flight deficits by adult-specific knockdown or abrogation of Ral/Exo84 (Richhariya et al., 2017; Figs. 1*D* and 3*B*) function agrees with an earlier finding where exocyst function was dispensable for synaptic release from mature neurons (Murthy et al., 2003).

In pupal dopaminergic neurons, Ral-modulated vesicle release is a likely mechanism by which SOCE affects synaptic maturation and thus function of adult flight circuit neurons. Regulation of circadian activity by Ral has been demonstrated in *Drosophila* (Klose et al., 2016). Our results suggest that such regulation may be initiated by receptor-stimulated SOCE. Moreover, SOCE-regulated Ral expression and vesicle release at presynaptic sites may act as co-incident detectors of neuromodulatory signals that enhance synaptic efficacy and network drive for optimal function of cognate neural circuits in other organisms as well, including vertebrates.

## Synthesis

Reviewing Editor: Margaret McCarthy, Univ of Maryland School of Medicine Decisions are customarily a result of the Reviewing Editor and the peer reviewers coming together and discussing their recommendations until a consensus is reached. When revisions are invited, a fact-based synthesis statement explaining their decision and outlining what is needed to prepare a revision will be listed below. The following reviewer(s) agreed to reveal their identity: Hiromu Tanimoto.

The manuscript improved after the revision by addressing reviewers' criticisms.

One last thing to clarify before publication is the measurement of basal fluorescence (comment 12 in the decision letter). The authors' claim on spontaneous release relies on the basal fluorescence, but as the absolute fluorescent level before applying any stimuli is not shown anywhere in the manuscript. As different levels of basal fluorescence may affect the speed of photobleaching. the measurement of “basal release” is not possible, and the claim must be moderated accordingly.

## Author Response

May 06, 2018

Dear Dr. McCarthy,

We are now ready to upload a revised version of our manuscript "dSTIM and Ral/exocyst mediated synaptic release from pupal dopaminergic neurons sustains Drosophila flight".

Both text and figures have been revised appropriately to address the concerns of reviewers. Details of the revisions made are described in the Response to Reviewers. For ease of the reviewers we have also appended a track change version of the manuscript. We hope that with these changes, the manuscript will be acceptable for publication.

Yours Sincerely,

Gaiti Hasan
