# Supplementary figures and images for: dSTIM- and Ral/Exocyst-Mediated Synaptic Release from Pupal Dopaminergic Neurons Sustains Drosophila Flight
Source: eNeuro. 2018 Jun 18;5(3):ENEURO.0455-17.2018. doi: 10.1523/ENEURO.0455-17.2018 (PMC6011419; doi:10.1523/ENEURO.0455-17.2018)

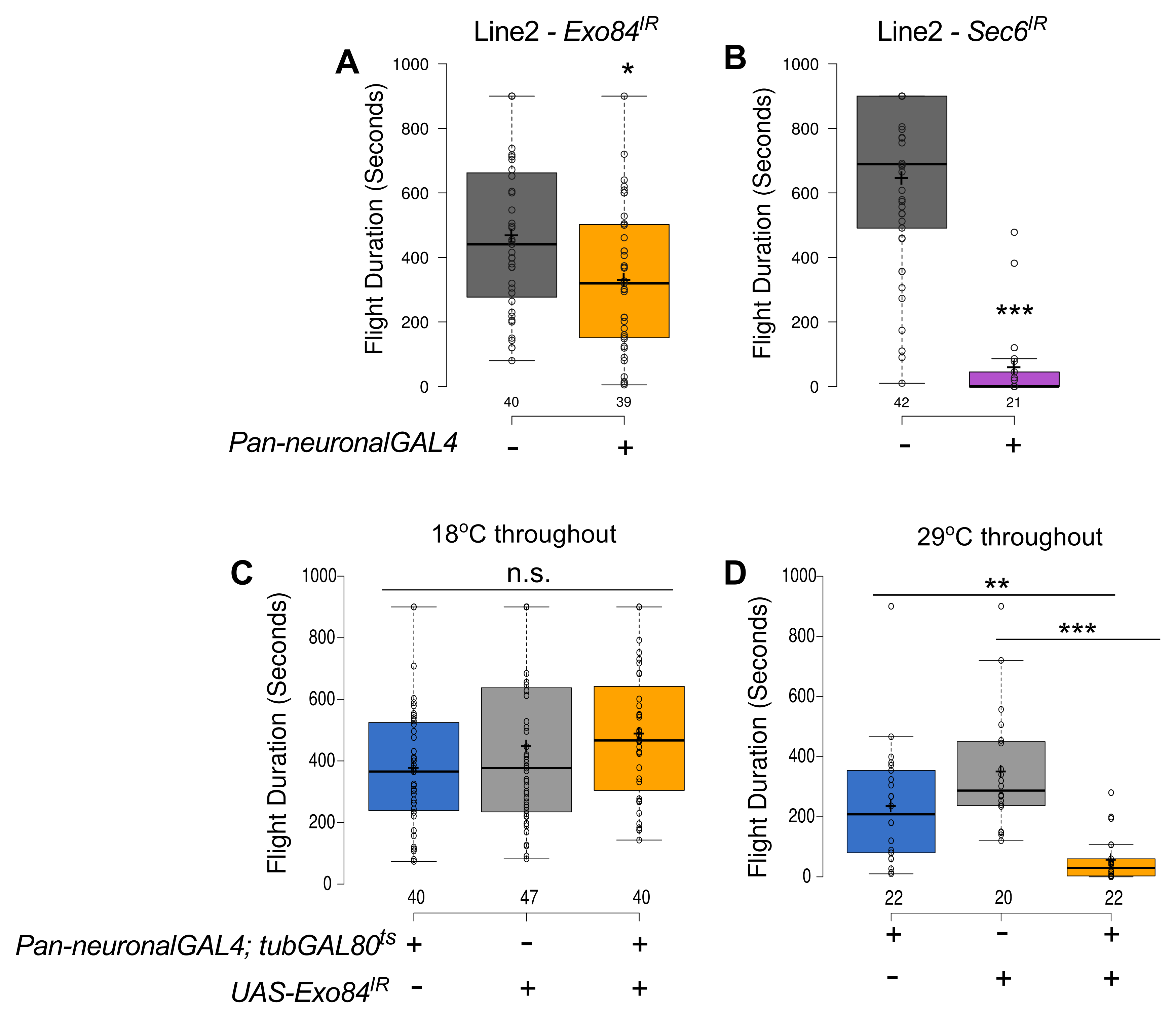

Supplement: Figure 1-1 — The role of exocyst components in flight. A, B, Box plots represent flight durations of flies of the indicated genotypes. Knockdown of Exo84 and Sec6 with alternate RNAi lines resulted in similar phenotypes as shown in Fig. 1. These flies were grown at 25°C throughout. C, D, Box plots represent flight durations of control flies with RNAi-mediated knockdown of Exo84 (Exo84IR) in neurons raised at the indicated temperatures throughout development. At the permissive temperature of 18°C, there is no knockdown. GAL4-mediated knockdown of Exo84 occurs at the restrictive temperature of 29°C. Box plot symbols are as described in Fig. 1. *, p < 0.05, **, p < 0.01, ***, p < 0.001, n.s., not significant at p < 0.05 by two-tailed Student’s t test (for A and B) or one-way ANOVA followed by post hoc Tukey’s test (for C and D). All comparisons for significance were with the control values except where marked by a horizontal line. For exact p-values, refer to Table 1-1. Download Figure 1-1, TIF file. [file sup_enu-eN-NWR-0455-17-s02.tif]

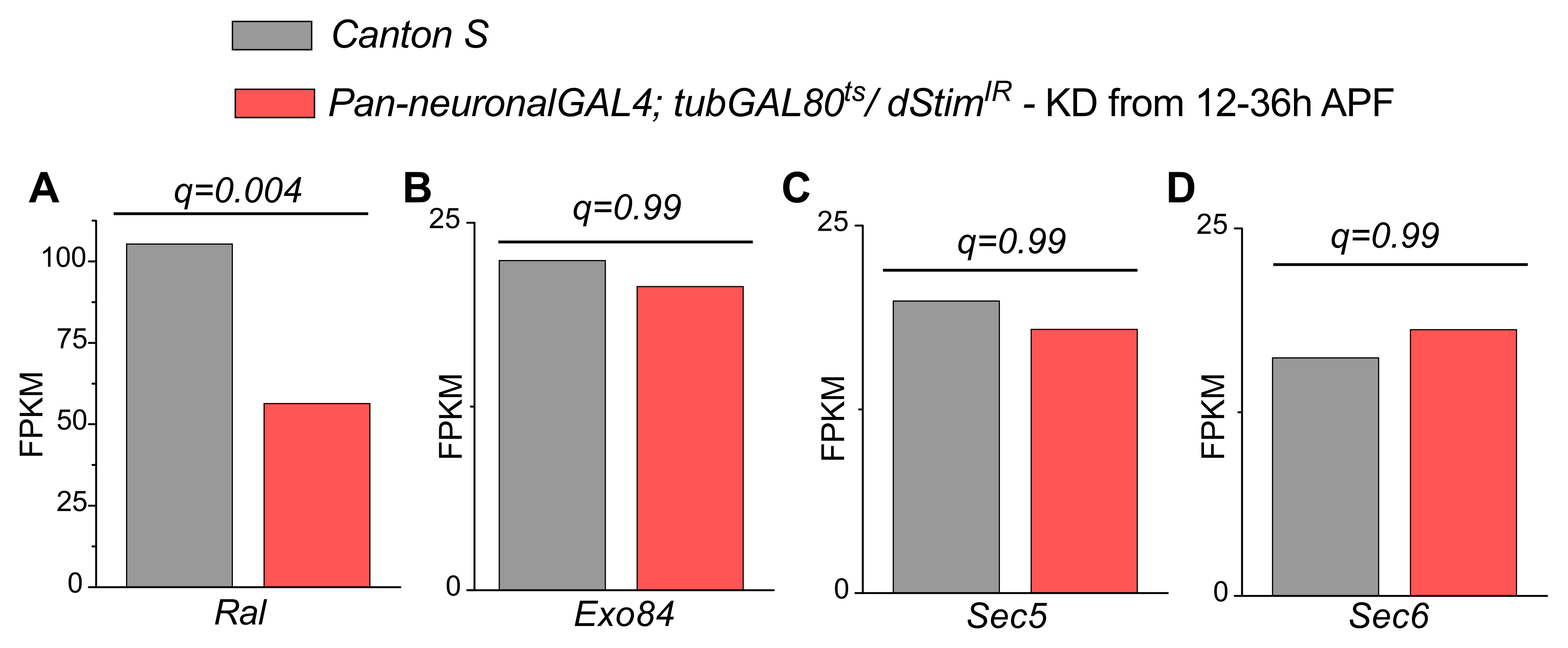

Supplement: Figure 1-2 — Expression of exocyst components is not regulated by SOCE. A–D, Bar graphs represent the level of expression of the indicated genes using values for fragments per kilobase gene per million reads (FPKM) in control and dStim knockdown pupal brains. These data were obtained from the transcriptomic screen performed in Richhariya et al. (2017). q-values are as obtained by CuffDiff. Download Figure 1-2, TIF file. [file sup_enu-eN-NWR-0455-17-s03.tif]

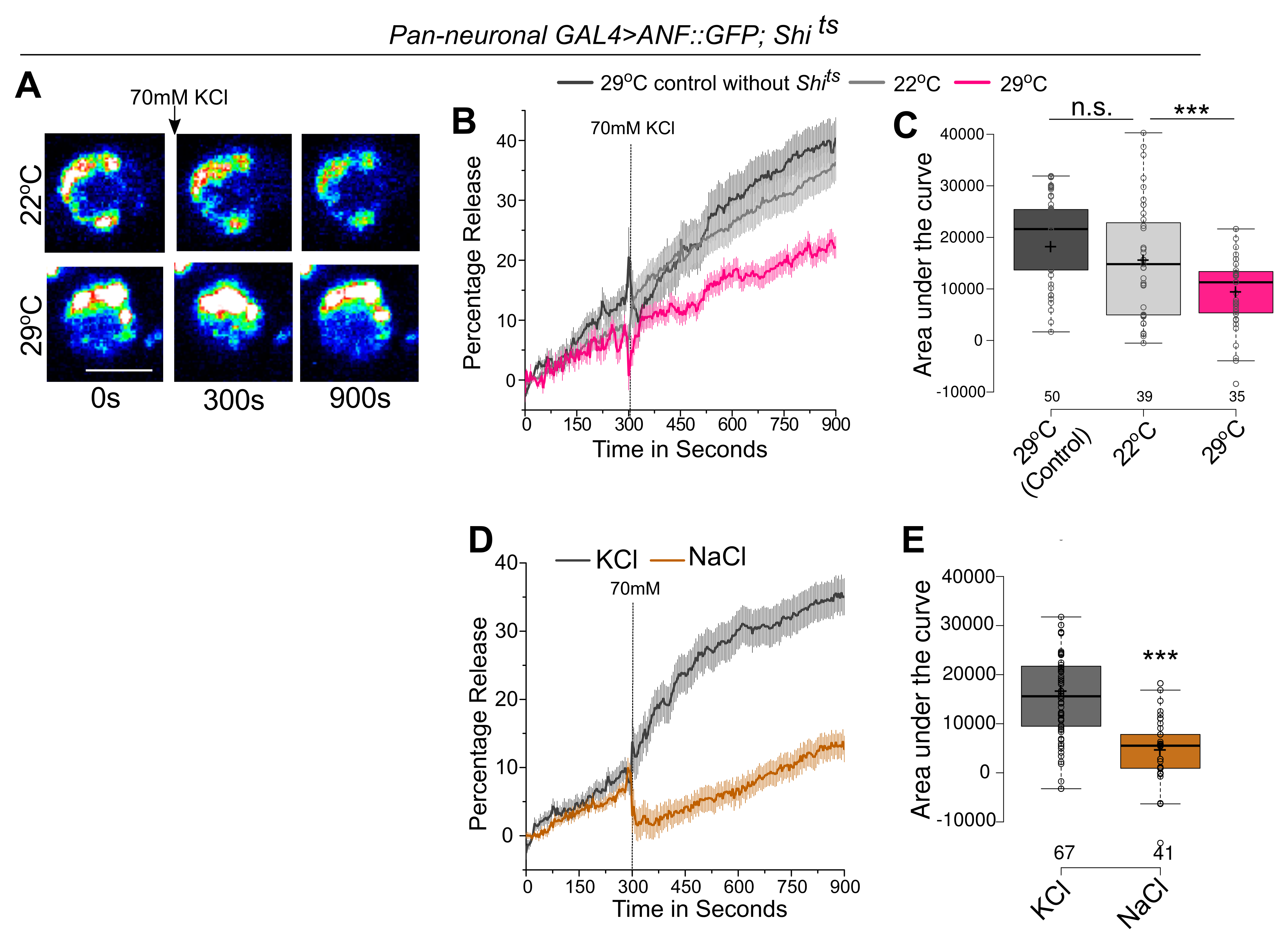

Supplement: Figure 2-1 — Assay to measure vesicular release from cultured pupal neurons. A, Representative images of single somata of cultured pupal neurons expressing ANF::GFP and Shits. The images were obtained over time upon depolarization with KCl at permissive (22°C) and restrictive (29°C) temperatures. Scale bar represents 10 µm. B, D, Traces represent release of ANF::GFP, estimated by the reduction in GFP fluorescence, upon depolarization with KCl, or addition of NaCl over time. The control trace in D is the same as that in Fig. 2A. C, E, Amount of release quantified as area under the curve from 300 to 900 s is represented as box plots for the indicated genotypes. Box plot symbols are as described in Fig. 1. ***, p < 0.001, n.s., not significant at p < 0.05 by one-way ANOVA followed by post hoc Tukey’s test (for C) or two-tailed Student’s t test (for E). All comparisons for significance were with the control values except where marked by a horizontal line. For exact p-values, refer to Table 1-1. Download Figure 2-1, TIF file. [file sup_enu-eN-NWR-0455-17-s04.tif]

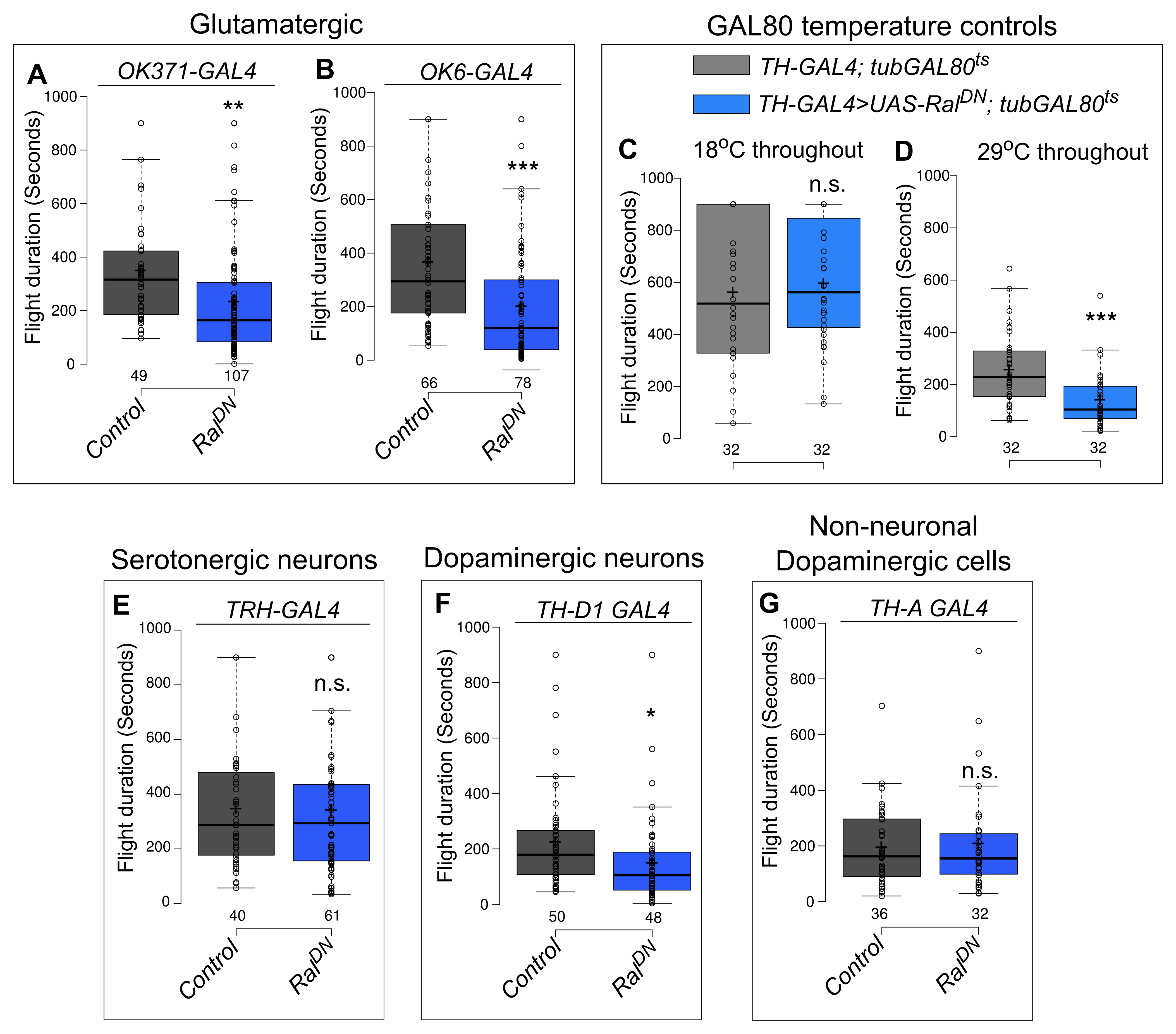

Supplement: Figure 3-1 — Neuronal subsets where Ral function is required for maintenance of flight bouts. A–G, Box plots represent flight durations of flies from the indicated genotypes measured by the single flight assay. Box plot symbols are as described in Fig. 1. Statistically distinguishable groups are indicated by different alphabets over the box plots (two-tailed Student’s t test, p < 0.05). *, p < 0.05, **, p < 0.01, ***, p < 0.001, n.s., not significant by two-tailed Student’s t test. For exact p-values, refer to Table 1-1. Download Figure 3-1, TIF file. [file sup_enu-eN-NWR-0455-17-s05.tif]

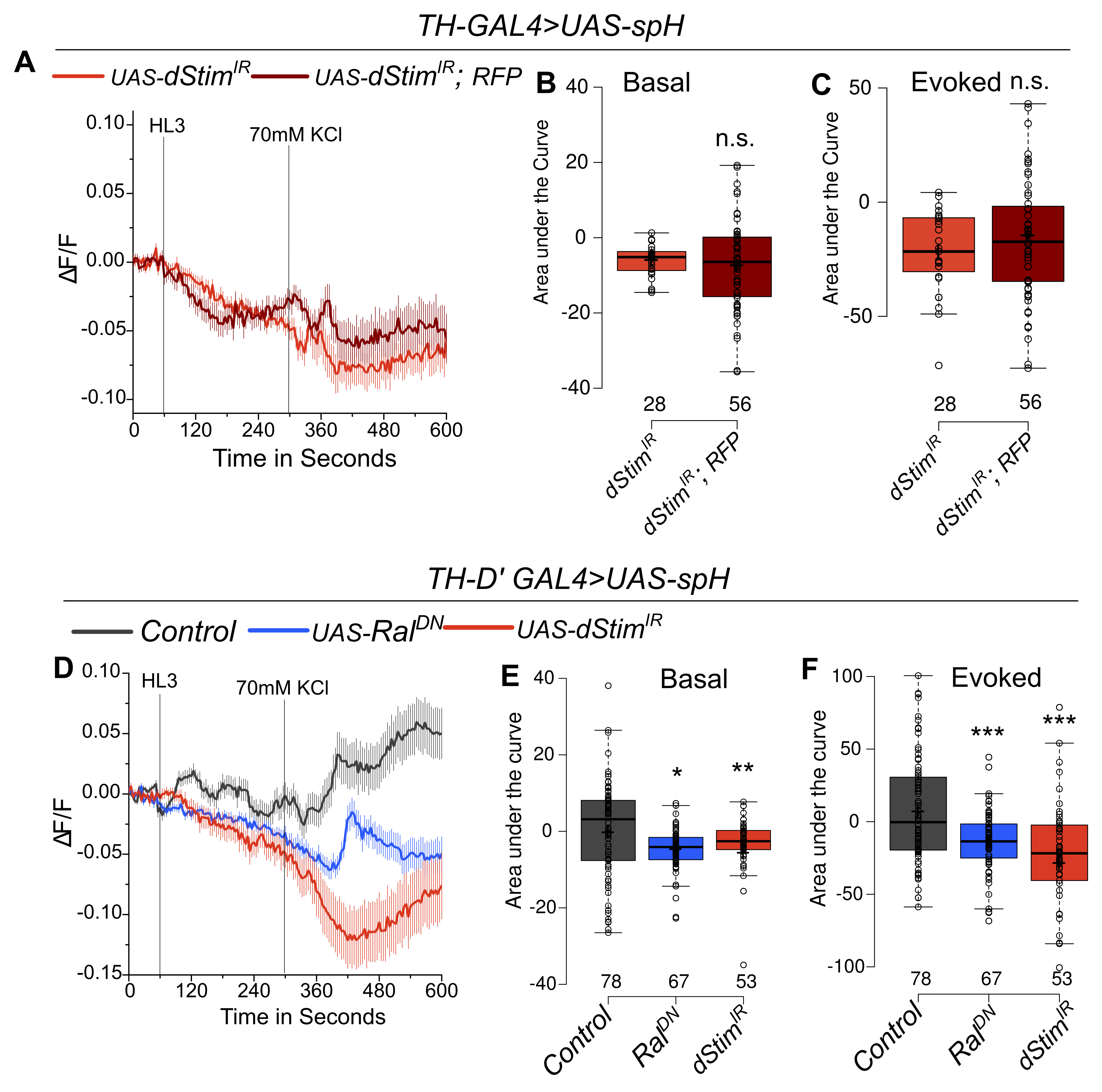

Supplement: Figure 4-1 — dStim and Ral regulate synaptic release from pupal dopaminergic neurons. A, D, Traces represent change in fluorescence of spH over time from brains of the indicated genotypes. Lines represent means and the error bars, standard error of means. Points of addition of HL3 and KCl are denoted by vertical lines. B, E, Saline-induced release as quantified by the area under the curve from 60 to 300 s of the respective traces. C, F, Evoked release as quantified by the area under the curve from 300 to 600 s of the respective traces. Box plot symbols are as described in Fig. 1. Numbers below the boxes represent the number of ROIs measured for change in fluorescence from a minimum of five brains per genotype. *, p < 0.05, **, p < 0.01, ***, p < 0.001, n.s., not significant at p < 0.05 by two-tailed Student’s t test (for B and C) or one-way ANOVA followed by post hoc Tukey’s test (for E and F). For exact p-values, refer to Table 1-1. Download Figure 4-1, TIF file. [file sup_enu-eN-NWR-0455-17-s06.tif]
